# Antioxidant enzymes and lipid peroxidation in endometrium of patients with polyps, myoma, hyperplasia and adenocarcinoma

**DOI:** 10.1186/1477-7827-7-149

**Published:** 2009-12-23

**Authors:** Snežana Pejić, Ana Todorović, Vesna Stojiljković, Jelena Kasapović, Snežana B Pajović

**Affiliations:** 1 Laboratory of Molecular Biology and Endocrinology, Vinča Institute of Nuclear Sciences, PO Box 522, 11001 Belgrade, Serbia

## Abstract

**Background:**

Oxidative stress and impaired antioxidant system have been proposed as a potential factors involved in the pathophysiology of diverse disease states, including carcinogenesis. In this study, we explored the lipid peroxidation levels and antioxidant enzyme activities in women diagnosed with different forms of gynecological diseases in order to evaluate the antioxidant status in endometrium of such patients.

**Methods:**

Endometrial tissues of gynecological patients with different diagnoses were collected and subjected to assays for superoxide dismutase, catalase, glutathione peroxidase, glutathione reductase and lipid hydroperoxides.

**Results:**

Superoxide dismutase activity was significantly decreased (50% in average) in hyperplastic and adenocarcinoma patients. Activities of both glutathione peroxidase and glutathione reductase were increased 60% and 100% on average, in hyperplastic patients, while in adenocarcinoma patients only glutathione reductase activity was elevated 100%. Catalase activity was significantly decreased in adenocarcinoma patients (47%). Lipid hydroperoxides level was negatively correlated to superoxide dismutase and catalase activities, and positively correlated to glutathione peroxidase and glutathione reductase activities.

**Conclusions:**

This study provided the first comparison of antioxidant status and lipid peroxidation in endometrial tissues of patients with polyps, myoma, hyperplasia and adenocarcinoma. The results showed that patients with premalignant (hyperplastic) and malignant (adenocarcinoma) lesions had enhanced lipid peroxidation and altered uterine antioxidant enzyme activities than patients with benign uterine diseases, polyps and myoma, although the extent of disturbance varied with the diagnosis. Further investigation is needed to clarify the mechanisms responsible for the observed alterations and whether lipid hydroperoxide levels and antioxidant enzyme activities in uterus of gynecological patients might be used as additional parameter in clinical evaluation of gynecological disorders.

## Background

It is now well known that free radicals and other reactive oxygen species (ROS) are continuously produced in all cells as a part of normal metabolism and in a wide variety of disease processes [1]. Deleterious effects of ROS and lipid peroxidation (LPO) products are counteracted by antioxidant (AO) defense system, which consists of nonenzymatic antioxidant molecules and antioxidant enzymes (AOE) such as superoxide dismutase (SOD), catalase (CAT), glutathione peroxidase (GPx), glutathione reductase (GR) and glutathione transferase (GST), [2].

Studies on the implication of oxidative stress in gynecologic disorders have not received adequate attention, and data regarding mechanisms underlying these diseases are sporadic. Findings suggest that maintenance of pro-antioxidant equilibrium is important in physiology of reproduction [3], embryopathies and pregnancy [4]. Clinical investigations indicate that women with certain common benign gynecological diseases, such as endometriosis and uterine leiomyoma, may have increased risk of developing malignant tumors [5]. Two of the most common malignancies of the female genital tract worldwide are cervical and endometrial cancer [6].

Classic clinicopathologic findings suggest that endometrial carcinoma arises through a series of precursor lesions known as endometrial hyperplasia [7]. According to the current World Health Organization (WHO) nomenclature, four categories of endometrial hyperplasia exist, simple (SH), complex (CH), simple atypical (SAH), and complex atypical (CAH). A new concept of classification (EIN nomenclature) has been proposed by Mutter et al. [8], based on integrated morphological, genetic, molecular, cell biological, and morphometrical studies, according to which three disease categories are discerned, benign hyperplasia, endometrial intraepithelial neoplasia (EIN) and cancer [9].

Although endometrial hyperplasia is regarded as a preliminary stage of endometrioid carcinoma [10], there is a lack of data on the relationship between oxidative stress and antioxidant enzymes in such patients. Some investigations so far have revealed elevated levels of lipid peroxidation and disturbed AOE activities in endometrial tissue of patients with benign and malign diseases. In patients with endometriosis and adenomyosis, SOD and CAT were found to be abnormally expressed [11,12], while patients with endometriosis have lowered GPx activity [13]. In endometrial cancer tissue of both Finnish and Japanese women, the activity of SOD was significantly lower than in normal endometrium, while GPx activity and LPO level were elevated [14]. Our recent results also show that gynecological patients have altered AO status in blood, which varies with the enzyme and diagnosis. However, both reduction in antioxidants and elevation of lipid peroxidation were observed in general [15].

Since study on this subject and in this area is still rare, we aimed to explore the level of lipid peroxidation and AOE activities in endometrium of patients diagnosed with myoma, polyps, hyperplasia simplex, hyperplasia complex and adenocarcinoma in order to evaluate the extent of oxidative stress in endometrial tissue of such patients.

## Methods

### Subjects

The material used in this study consisted of 88 tissue specimens of women admitted to the Department of Gynecology and Obstetrics for gynecological evaluation within routine checkups or for abnormal uterine bleeding. The specimens were taken after obtaining the informed consent and the study was conducted prospectively. The protocol was consistent with the World Medical Association Declaration of Helsinki (Ethical Principles for Medical Research Involving Human Subjects).

On the basis of diagnosis and histological examination, subjects were divided into following groups: patients with benign uterine changes (representing the control group of patients with an assumed unaffected endometrium): polypus endometrii (PE, n = 18, 45 ± 3 yr) or uterine myomatosis (UM, n = 12, 47 ± 2 yr); patients with abnormal bleeding: hyperplasia simplex endometrii (SH, n = 31, 48 ± 1 yr), hyperplasia complex endometrii (CH, n = 22, 48 ± 2 yr) or adenocarcinoma endometrii, stage I (ACE, n = 5, 59 ± 3 yr).

### Samples

After curettage performed under general anesthesia, endometrial tissue samples were washed immediately in saline solution and transferred to the laboratory. Upon receipt of samples they were homogenized in phosphate buffer containing 0.05 M KH_2_PO_4 _and 1 mM EDTA, pH 7.8 (1 g tissue per 2 ml buffer) in a Teflon/glass homogenizer (Spindler & Hoyer, Göttingen, Germany) aliquoted and frozen at -70°C for 20 h in order to disrupt cell membranes. For SOD assay (OxisResearch™), thawed homogenates were vortexed 1 min and centrifuged at 8600 g, for 20 min at 4°C (Eppendorf centrifuge 5417, Eppendorf AG, Hamburg, Germany). According to manufacturer's recommendation, after addition of ethanol/chloroform extraction reagent (62.5/37.5 vol/vol) to completely remove hemoglobin interference, samples were centrifuged at 6000 g for 20 min, at 4°C (Beckman centrifuge J2-21, Beckman Instruments Inc., Palo Alto, CA, USA). Upper aqueous layer was collected and kept at -70°C until assay. The enzyme activities and lipid hydroperoxide (LOOH) concentration were monitored spectrophotometrically (Perkin Elmer Spectrophotometer, Lambda 25, Perkin Elmer Instruments, Norwalk, CT, USA). Samples were kept frozen and measurements were performed within 1 month upon their receipt. The specific enzyme activities were expressed as Units (U) or mU per milligram of total cell protein (U or mU/mg protein). LOOH concentration was expressed as nmol/mg protein. Determination of protein concentration was performed in tissues homogenates by the method of Lowry et al. [16] and expressed as mg/ml.

### Assays

#### Assay of SOD activity

Determination of SOD activity was performed using Oxis Bioxytech^® ^SOD-525™ Assay (Oxis International, Inc., Portland, OR, USA). The method is based on SOD-mediated increase of autoxidation of 5,6,6a11b-tetrahydro-3,9,10-tryhydroxybenzo [c]fluorene in aqueous alkaline solution to yield a chromophore with maximum absorbance at 525 nm. The SOD activity is determined from the ratio of the autoxidation rates in the presence (Vs) and in the absence (Vc) of SOD. One SOD-525 activity unit is defined as the activity that doubles the autoxidation rate of the control blank.

#### Assay of CAT activity

CAT activity was determined by the method of Beutler [17]. The reaction is based on the rate of H2O2 degradation by catalase contained in the examined samples. The reaction was performed in an incubation mixture containing 1 M Tris-HCl, 5 mM EDTA, pH 8.0, and monitored spectrophotometrically at 230 nm. One unit of CAT activity is defined as 1 μmol of H2O2 decomposed per minute under the assay conditions.

#### Assay of GPX activity

GPx activity was assessed using the Oxis Bioxytech^® ^GPx-340™ Assay (Oxis International, Inc., Portland, OR, USA), based on the principle that oxidized glutathione (GSSG) produced upon reduction of an organic peroxide by GPx, is immediately recycled to its reduced form (GSH) with concomitant oxidation of NADPH to NADP+. The oxidation of NADPH was monitored spectrophotometrically as a decrease in absorbance at 340 nm. One GPx-340 unit is defined as 1 μmol of NADH oxidized per minute under the assay conditions.

#### Assay of GR activity

Activity of GR was measured using the Oxis Bioxytech^® ^GR-340™ Assay (Oxis International, Inc., Portland, OR, USA). Assay is based on the oxidation of NADPH to NADP+ during the reduction of oxidized glutathione (GSSG), catalyzed by a limiting concentration of glutathione reductase. The oxidation of NADPH was monitored spectrophotometrically as a decrease in absorbance at 340 nm. One GR-340 unit is defined as 1 μmol of NADH oxidized per minute under the assay conditions.

#### Lipid hydroperoxides

Concentration of LOOH was measured by Oxis Bioxytech^® ^LPO-560™ Assay (Oxis International, Inc., Portland, OR, USA), which is based on the oxidation of ferrous (Fe^2+^) ions to ferric (Fe^3+^) ions by hydroperoxides under acidic conditions. Ferric ions then bind with the indicator dye, xylenol orange, and form a colored complex. The absorbance of the complex was measured at 560 nm. Since hydrogen peroxide content in many biological samples is much higher than that of other hydroperoxides, samples were pretreated with catalase to decompose the existing H_2_O_2 _and eliminate the interference.

### Statistical analysis

Statistical analysis was carried out by use of the nonparametric Kruskal-Wallis test and the Dunn's *post hoc test*, which considered the unequal and small sample sizes we used in this study (values which are not sharing the same letter are significantly different, p < 0.05). Relative values for LOOH level and enzyme activities were expressed as percent of corresponding values in patients with polyps, which are considered to be 100%. Spearman's rank correlation coefficient was used to investigate associations between lipid peroxidation and antioxidant enzyme activities. Two-tailed *p *values are given throughout. All data were analyzed using GraphPad Prism 4 software.

## Results

Comparing data for the level of lipid hydroperoxides and antioxidant enzyme activities among five diagnosis groups, all parameters showed significant variations of the obtained values (LOOH: H = 37.11, df = 4, p < 0.001; SOD: H = 31.72, df = 4, p < 0.001; CAT: H = 11.27, df = 4, p < 0.05; GPx: H = 26.54, df = 4, p < 0.001; GR: H = 29.22, df = 4, p < 0.001; Kruskal-Wallis).

**Figure 1:**
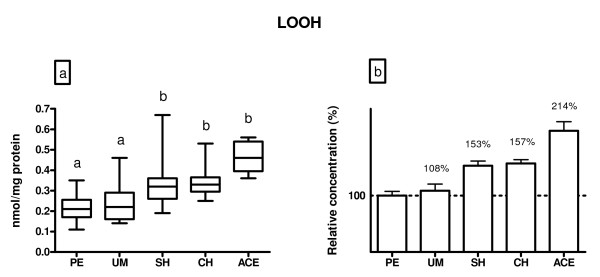
**A) LOOH concentration (nmol/mg protein) in endometrium of patients diagnosed with: polypus endometrii (PE), uterine myomatosis (UM), simple hyperplasia (SH), complex hyperplasia (CH) and adenocarcinoma endometrii (ACE)**. Mean LOOH concentrations (± SD) are represented by the box; medians are plotted inside a box; the whiskers extend to the 5th and 95th percentiles. Values which are not sharing the same letter (a, b) are significantly different (p < 0.05). B) Relative values of LOOH concentration in the examined diagnosis, where PE was taken as 100%.

Figure 1 indicates the lipid peroxidation levels in the examined groups of patients. Patients with myoma had comparable LOOH level to that observed in patients with polyps, whereas it was significantly higher in patients with simple or complex hyperplasia (50%, p < 0.05) and adenocarcinoma (105%, p < 0.05), compared to polyps and myoma subjects.

**Figure 2:**
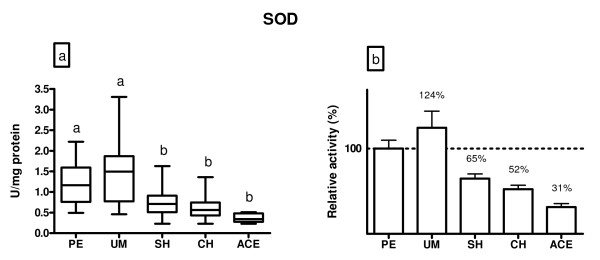
**A) SOD activity (U/mg protein) in endometrium of patients diagnosed with: polypus endometrii (PE), uterine myomatosis (UM), simple hyperplasia (SH), complex hyperplasia (CH) and adenocarcinoma endometrii (ACE)**. Mean SOD activities (± SD) are represented by the box; medians are plotted inside a box; the whiskers extend to the 5th and 95th percentiles. Values which are not sharing the same letter (a, b) are significantly different (p < 0.05). B) Relative values of SOD activity in the examined diagnosis, where PE was taken as 100%.

Superoxide dismutase activity is shown in Figure 2. The SOD activity was not different between polyps and myoma patients (p > 0.05). Compared to subjects with benign endometrial changes (polyps and myoma), SOD activity was significantly decreased in patients with hyperplasia simplex (41%, p < 0.05), hyperplasia complex (53%, p < 0.05) and adenocarcinoma (72%, p < 0.05) patients.

**Figure 3:**
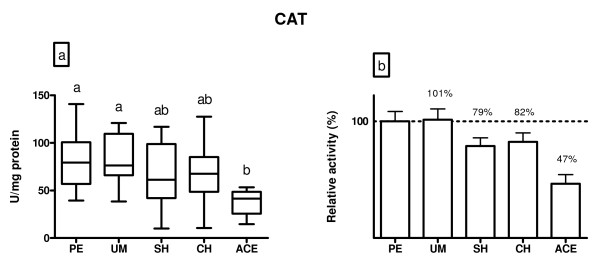
**A) CAT activity (U/mg protein) in endometrium of patients diagnosed with: polypus endometrii (PE), uterine myomatosis (UM), simple hyperplasia (SH), complex hyperplasia (CH) and adenocarcinoma endometrii (ACE)**. Mean CAT activities (± SD) are represented by the box; medians are plotted inside a box; the whiskers extend to the 5th and 95th percentiles. Values which are not sharing the same letter (a, b) are significantly different (p < 0.05). B) Relative values of CAT activity in the examined diagnosis, where PE was taken as 100%.

Catalase activity is shown in Figure 3. The activity was not altered between polyps and myoma patients. Compared to polyps and myoma subjects, patients with simple and complex hyperplasia did not have significantly lower CAT activity (p > 0.05), while in adenocarcinoma patients the activity was lower by 43% (p < 0.05).

**Figure 4:**
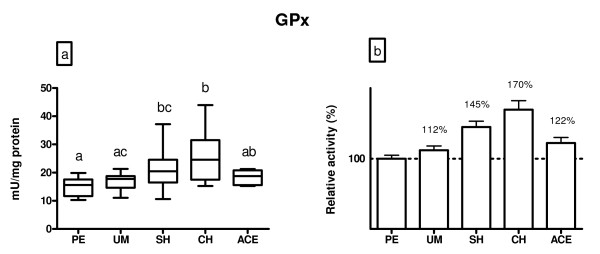
**A) GPx activity (mU/mg protein) in endometrium of patients diagnosed with: polypus endometrii (PE), uterine myomatosis (UM), simple hyperplasia (SH), complex hyperplasia (CH) and adenocarcinoma endometrii (ACE)**. Mean GPx activities (± SD) are represented by the box; medians are plotted inside a box; the whiskers extend to the 5th and 95th percentiles. Values which are not sharing the same letter (a, b, c) are significantly different (p < 0.05). B) Relative values of GPx activity in the examined diagnosis, where PE was taken as 100%.

Figure 4 shows glutathione peroxidase activity. Activity of this enzyme was not different between polyps and myoma patients (p > 0.05). Patients with hyperplasia simplex had 45% (p < 0.05) higher GPx activity than patients with polyps. Women with complex hyperplasia had 61% (p < 0.05) higher GPx activity than women with polyps or myoma. There was no difference in GPx activity in adenocarcinoma patients in comparison to polyps or myoma patients (p > 0.05).

**Figure 5:**
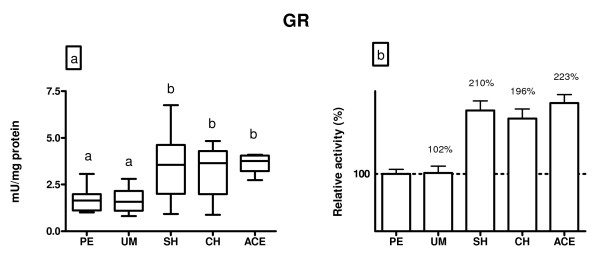
**A) GR activity (mU/mg protein) in endometrium of patients diagnosed with: polypus endometrii (PE), uterine myomatosis (UM), simple hyperplasia (SH), complex hyperplasia (CH) and adenocarcinoma endometrii (ACE)**. Mean GR activities (± SD) are represented by the box; medians are plotted inside a box; the whiskers extend to the 5th and 95th percentiles. Values which are not sharing the same letter (a, b) are significantly different (p < 0.05). B) Relative values of GR activity in the examined diagnosis, where PE was taken as 100%.

Glutathione reductase activity is shown in figure 5. Compared to patients with polyps and myoma, significant elevation of GR activity was recorded in all examined groups, hyperplasia simplex (108%, p < 0.05), hyperplasia complex (94%, p < 0.05) and adenocarcinoma (120%, p < 0.05).

**Figure 6:**
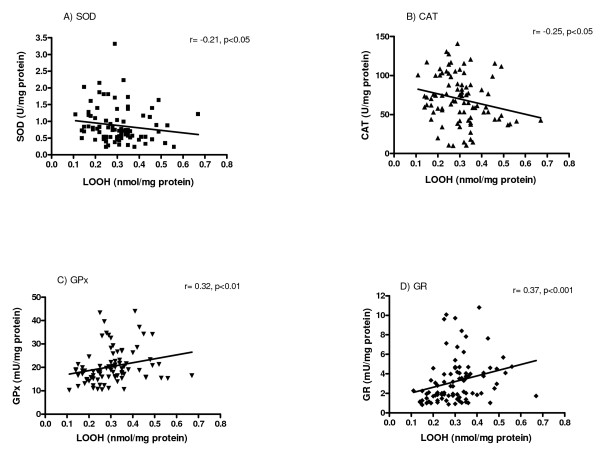
**Scatter plot of SOD (A), CAT (B), GPx (C), and GR (D) activities against LOOH level**.

Correlation analysis (Figure 6) showed that lipid hydroperoxides level was negatively correlated to SOD (r = -0.21, p < 0.05) and CAT activity (r = -0.25, p < 0.05), and positively correlated to GPx activity (r = 0.32, p < 0.01) and GR activity (r = 0.37, p < 0.001) (Figure 6). Although the correlation coefficient values were low (indicative of a weak correlation), the correlation was supported by the p-values showing statistical significance.

## Discussion

Oxidative stress and impaired AO system have been proposed as a potential factors involved in the pathophysiology of diverse disease states, including carcinogenesis. This study indicates that women with gynecological disorders have altered activity of antioxidant enzymes and it also points to elevated levels of lipid peroxidation products, as markers of oxidative stress, in endometrial tissue of such patients. Namely, higher levels of LOOH were recorded in patients with endometrial hyperplasia or adenocarcinoma than in patients with benign (polyps) or presumably unaffected (myoma) endometrial tissue. Patients with adenocarcinoma had higher LOOH level than the hyperplastic ones, while there was no difference in LOOH level between women with benign diagnoses.

Our findings are consistent with previous studies suggesting that elevation of LOOH level might reflect the impaired oxidant/antioxidant balance in favor of oxidants and oxidative stress [14,18]. It could also be associated with degenerative changes in morphological and physiological features of endometrial tissue. It is known that most normal tissues with high cellular turnover (like endometrium) utilize circulating lipids preferentially for the synthesis of new structural lipids [19-21], while hyperplastic and neoplastic tissues seem to necessitate the additional sources for more energetic storage, like increased synthesis of endogenous fatty acids [22,23]. The fatty acid pathway might promote localized oxidative stress in hyperplastic tissue and highly proliferative lesions [24].

Superoxide dismutase activity was found to be decreased in endometrium of patients with hyperplasia and adenocarcinoma in comparison to women with polyps or myoma. In patients with adenocarcinoma, the decrease in SOD activity was more pronounced than in hyperplastic subjects. Similar results were obtained in endometrial cancer tissue of both Finnish and Japanese women [14]. The decrease in enzyme activity appeared to be related to the decreased levels of SOD protein in the examined tissues [25]. Also, it may be due to an increased endogenous production of ROS as evidenced by increased lipid hydroperoxides. *In vitro *studies indicate that all SOD enzymes are exquisitely susceptible to oxidative modification and inactivation through targeting of tyrosine or histidine residues [26,27]. The SOD activity observed in patients with polyps and myoma may also point to a role of oxidative stress in genesis of precancerous lesions and cancer. Namely, studies suggest that women with certain common benign gynecological diseases, such as endometriosis, leiomyomas or polyps may experience increased risks of developing malignant tumors [5,28,29]. Research also suggests that transformed tissues produce high levels of ROS and are constantly under oxidative stress [30]. The increase in ROS, such as O_2 _^.-^, is able to stimulate cell cycle progression and promote cell proliferation.

Activity of CAT was significantly decreased in adenocarcinoma patients when compared to women with polyps or myoma. Data regarding CAT in uterine pathology are different. Punnonen et al. [14] found that Japanese women with endometrial cancer did not have significantly altered CAT activity. Study of Ota et al. [12] showed that women with endometriosis and adenomyosis had elevated expression of CAT in comparison to healthy women but the expression did not show the usual, fluctuating pattern characteristic for the menstrual cycle. Lower CAT activity, observed in our study, might enhance the oxidative stress in patients by lower detoxification of H_2_O_2_. Such conditions may accelerate the cell transformation and, by that, the tumor growth. It is already known that if the O_2 _^.- ^is not removed immediately, it may cause the inhibition of CAT activity [31]. Thus, the lower CAT activity we recorded might be a consequence of the decreased SOD activity in hyperplastic and cancer patients. In addition, we found negative correlation between LOOH level and CAT activity in the examined patients. It is possible that such relations contribute to a certain antiapoptotic milieu that is suitable for enhancing the mutations frequency, a condition which may lead to the cell transformation and cancer. Investigations already point to that mechanism [32].

In comparison with polyps or myoma subjects, GPx activity was increased in patients with hyperplasia. This elevation was followed by the increase in the GR activity. A positive correlation between LOOH level and GPx and GR activities in endometrium was also recorded. Much research so far has been focused on deleterious effects of elevated oxygen and nitrogen species as an integral part of the pathophysiology of many diseases. However, the precise role of hydroperoxides, as well as of GPx, in gynecological pathology remains to be determined. Recent studies indicate that peroxidative stress is one basis for the pathogenesis of inflammation-associated cancers [33,34]. One of such predisposing conditions closely related to inflammation and the development of ovarian cancer is endometriosis [35,36]. Punnonen et al. [14] detected higher GPx activity in cancer than in normal endometrium of the Finnish population while in Japanese women no change of GPx activity was recorded. Ohwada et al. [37] also observed higher GPx activity in endometrial cancer tissue than that from the healthy controls. Moreover, they found significantly higher GPx activity in histological grade 1 endometrial cancer than in endometrial cancer of histological grades 2 or 3. GPx activity was also significantly higher in endometrial cancer with mixed atypical adenomatous endometrial hyperplasia than in endometrial cancer without endometrial hyperplasia [37]. In population of Spanish women with epithelial ovarian carcinoma, Sanchez et al. [38] also observed a significantly increased GPx activity compared with control normal tissue.

Evidence suggests a protective role of overexpressed [39] or activated [40,41] GPx activity by various environmental stresses including cancer. However, elevated GPx gene expression has also been associated with tumorogenesis, presumably because of its antiapoptotic activity [42-44]. Previous analysis of the histological and clinical features of endometrial cancer showed that high levels of GPx activity were associated with well-differentiated adenocarcinoma, slight myometrial invasion, and the presence of concurrent endometrial hyperplasia. These observations suggested that women with endometrial cancer who had a high level of GPx activity in their endometrial tissue were more likely to have a good prognosis; that is, GPx activity may be a significant prognostic factor for endometrial cancer [37].

## Conclusions

In summary, this study showed that patients with premalignant (hyperplastic) and malignant (ACE) lesions had enhanced lipid peroxidation and altered uterine antioxidant enzyme activities than patients with benign uterine diseases, polyps and myoma, although the extent of disturbance varied with the diagnosis. Generally, it is not unknown that AO defense system is altered in response to various diseases and that oxidative stress may be a common pathogenic mechanism implicated in the pathogenesis of diverse disease states, including gynecological disorders. Although our results do not allow us to draw a conclusion whether the recorded changes in AOE activities in hyperplastic and carcinoma patients are the cause or the consequence of increased oxidative stress, they provided the first comparison of AO status and lipid peroxidation in patients with uterine polyps, myoma, hyperplasia and adenocarcinoma. Further investigation should determine whether lipid hydroperoxide levels and AOE activities in uterus of gynecological patients might be used as additional parameters in clinical evaluation of the stage of gynecological disorders.

## Competing interests

The authors declare that they have no competing interests.

## Authors' contributions

SP carried out the study, performed the statistical analysis and drafted the manuscript. AT, VS, and JK participated in the sample preparation and they contributed to perform the assays. SBP was responsible for clinical data acquisition, management and supervision. All authors read and approved the final manuscript.
